# Hernia 3D training model: a new inguinal hernia 3D-printed simulator

**DOI:** 10.31744/einstein_journal/2024AO0620

**Published:** 2024-07-29

**Authors:** Paulo Henrique Fogaça de Barros, Camila Scivoletto Borges, Caroline Petersen da Costa Ferreira, Bruno de Lucia Hernani, Iron Pires Abreu, Luciano Tastaldi, Leandro Totti Cavazzola

**Affiliations:** 1 Hospital Alemão Oswaldo Cruz São Paulo SP Brazil Hospital Alemão Oswaldo Cruz, São Paulo, SP, Brazil.; 2 Centro Universitário FMABC Santo André SP Brazil Centro Universitário FMABC, Santo André, SP, Brazil.; 3 University of Texas Medical Branch Galveston TX USA University of Texas Medical Branch, Galveston, TX, USA.; 4 Hospital de Clínicas de Porto Alegre Porto Alegre RS Brazil Hospital de Clínicas de Porto Alegre, Porto Alegre, RS, Brazil.

**Keywords:** Hernia, inguinal, Minimally invasive surgical procedures, Printing, three-dimensional, Simulation training

## Abstract

**Objective:**

To evaluate the functionality and quality of the anatomical representation of the hernia 3D training model.

**Methods:**

A model was created based on subtraction data derived from computed tomography scans of the pelvic bones and lumbar spine using the Blender 3.2.2 software program. Images were modeled and reconstructed in 3D to display the male inguinal region, typically viewed using a laparoscopic approach. Polylactic acid plastic was used to print the model. Some structures were made using ethylene vinyl acetate to enable possible material replacement and model reutilization. Thirty surgeons with various training levels were invited to use the model. Transabdominal inguinal hernioplasty was performed by simulating the same steps as those of a laparoscopic surgery, and the surgeons answered a questionnaire regarding the simulation.

**Results:**

Twenty-eight surgeons responded, seven of whom were experts in the treatment of abdominal wall hernias. The model was deemed easy to use, realistic, and anatomically precise, establishing it as a valuable supplement to minimally invasive surgery training.

**Conclusion:**

The evaluation of this 3D model was favorable, as it accurately depicted the inguinal region anatomically, while also proving to be cost-effective for training purposes. The model could be a good option, particularly beneficial for training surgeons at the beginning of their careers.

## In Brief

Barros et al. demonstrated a 3D printed model that exhibits anatomical representativeness, low cost, and scalability. The model was created based on subtraction data obtained from computed tomography scans. Images were modeled and reconstructed in 3D to display the male inguinal region, typically viewed using a laparoscopic approach.

## Highlights

The model was considered simple, realistic, and capable of precisely simulating inguinal anatomy.

Recreating the inguinal region in a more anatomical manner without increasing costs is possible.

The model could be a good option, particularly beneficial for training surgeons at the beginning of their careers.

## Graphical abstract

**Figure:**
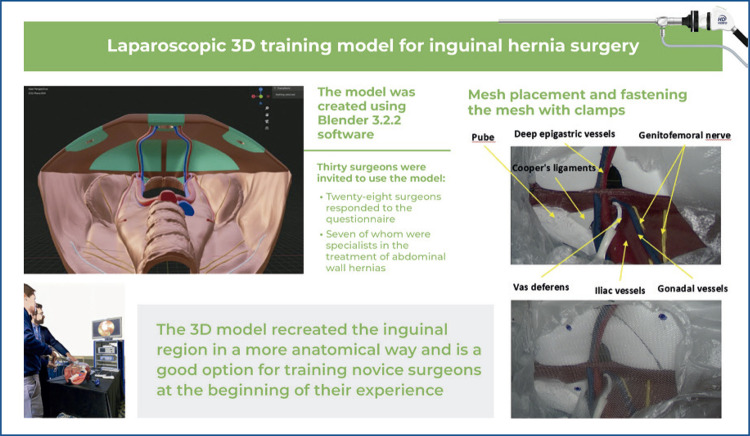

## INTRODUCTION

Inguinal hernia (IH) is a common complication of abdominal surgery. Approximately 20 million patients worldwide require surgical repair annually.^(1,2)^ In the United States, the use of minimally invasive surgery (MIS) has increased in recent years.^(3)^ Despite having a similar recurrence rate to that of the Lichtenstein technique, MIS results in less postoperative pain and earlier return to normal daily activities.^1^

The main challenges of MIS are its long learning curve and the unrealistic simulation resources available for training.^(5)^ One of the main risk factors for IH recurrence after surgery is inadequate training.^(1)^ When inquired about their previous experience, most surgical residents mentioned inadequate training and a difficult learning curve as obstacles to adopting laparoscopic techniques.^(5)^

Different training formats exist for developing these skills, ranging from virtual platforms to supervised operations. Model training has been shown to improve surgical results, reduce the length of stay, and shorten the operative time.^(6)^ Furthermore, it fosters training opportunities in a safe environment, thereby decreasing patient risks^(5)^ and promoting improvements in the surgical learning curve.

The cost of training presents another important obstacle, given that both virtual simulators and the use of cadavers are expensive. Although several low-cost models are available for simulating the inguinal region, they provide low-quality anatomical representations. This study demonstrated a model created using a three-dimensional (3D) printer.

## OBJECTIVE

To evaluate the functionality and quality of the anatomical representation of the hernia 3D training model.

## METHODS

### Creation of the model

**Figure 1:**
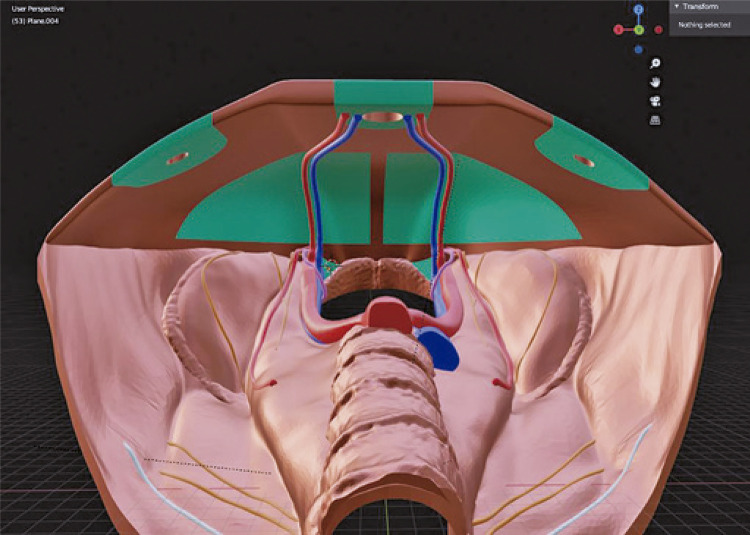
3D modeling in Blender (version 3.2.2) software based on the videolaparoscopic view

**Figure 2:**
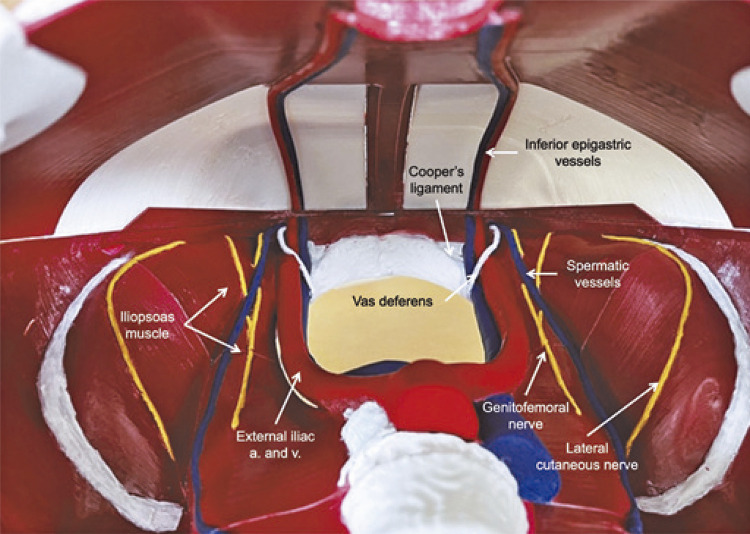
Painted view (for didatic purpose) of 3D printing model using polylactic acid plastic

The model was created based on subtraction data obtained from computed tomography scans of the pelvic bones and lumbar spine using the Blender 3.2.2 software program. Images were modeled and reconstructed in 3D to display the male inguinal region, typically visualized using a laparoscopic approach (Figure 1). Initially, the quadratus lumborum and iliopsoas muscles were recreated, adhering to their insertions on the pelvic and lumbar spinal bones. Subsequently, the iliac, deep epigastric, and gonadal vessels, as well as the vas deferens, were inserted. The inguinal nerves were displayed, adhering to their anatomical limitations. Polylactic acid (PLA) plastic was used to print the model. The model was painted to improve the training didactics (Figure 2). Some structures were prepared using ethylene vinyl acetate (EVA) to enable possible material replacement and model reutilization. Peritoneum was recreated using GLAD and PRESS’N SEAL^Ò^ Plastic wrap (Press’n Seal; Glad, Oakland, CA). The file for 3D printing is available in the Supplementary Materials (Online Resource). The estimated price for reproducing the model is two hundred and fifty Brazilian Reals (250 BRL), approximately fifty American Dollars (50 USD), as well as the requirement of a 3D printer.

### Participants

**Figure 3:**
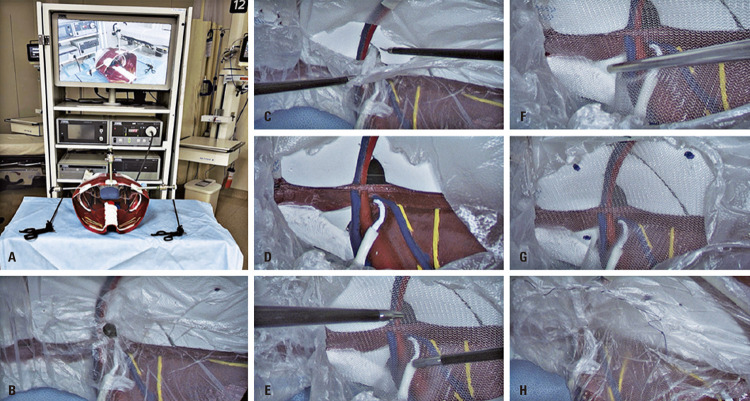
Placement of trocars (A); right lateral inguinal hernia (B); peritoneal opening and reduction of the herniary sac (C and D); mesh placement (E); fastening the mesh with clamps (F and G); and suturing the closure of the peritoneum (H)

Thirty surgeons with different training levels and expertise in MIS repairs were invited to use the model. The Institutional Review Board of *Hospital Alemão Oswaldo Cruz* approved the study procedures (CAAE: 62710322.9.0000.0070; #5.658.841), and all participants signed consent forms. The same surgical video equipment and instruments were used at the surgical simulation center. All surgeons performed a transabdominal preperitoneal procedure (TAPP) for IH repair, simulating the same surgical steps: trocar placement, peritoneal flap creation, hernial sac reduction, mesh placement and fixation, and peritoneal flap closure using sutures (Figure 3).

After the procedure, each participant completed a questionnaire to evaluate the model. They anonymously answered questions regarding the simulator and its importance in training.^(7,8)^ A 5-point Likert scale was used for the answers, and the data were stored in RedCap.

### Statistical analysis

The questionnaire contained items requiring rating on a 5-point Likert scale. Statistical analyses were performed using the RedCap platform. Median values were evaluated because the sample size was small, and the results were not normally distributed.

## RESULTS

Thirty surgeons agreed to participate and 28 answered the questionnaire. Table 1 lists the surgeons’ characteristics.

**Table 1:** Demographic data

Age (age range) (years)	36	(25–58)
Sex		
Female	10	(35.7)
Male	18	(64.3)
Experience in video laparoscopic surgery		
<50	8	(28.6)
50–100	4	(14.3)
>100	16	(57.1)
Experience in herniorrhaphy TAPP		
<10	18	(64.3)
10–30	1	(3.6)
>30	9	(32.1)

Values in the table are presented as the average (range) or n (%).

TAPP: transabdominal preperitoneal procedure.

The results on model opinions are presented in table 2. When asked about the model’s potential impact on education and training in inguinal herniorrhaphy, the surgeons completely agreed (with a median score of 5). Complete agreement (with a median score of 5) was also observed for resident training before entering the operating room.

**Table 2:** Pos- training feedback

Is the model completely realistic?	4 (3–5)
Does the model simulate human anatomy?	4 (3–5)
Can the model simulate peritoneal closure?	4 (1–5)
Can the model simulate dissection and mobilization?	4 (2–5)
Is this simulator easy to use?	4 (3–5)
Would I use the simulator in the hospital if it were available?	4.5 (3–5)
If I had this simulator, would I use it daily to practice?	4 (2–5)
Is it useful for educating professionals on inguinal herniorrhaphy TAPP?	5 (4–5)
Is it useful for inguinal herniorrhaphy TAPP training?	5 (4–5)
Is this simulator valuable for training residents before entering the operating room?	5 (4–5)
Should this simulator be introduced in the residency curriculum?	4 (3–5)

Likert scale: 1, completely disagree; 2, disagree; 3, neutral; 4, agree; 5, completely agree.

TAPP: transabdominal preperitoneal procedure.

## DISCUSSION

Minimally invasive surgery has demonstrated advantages for repairing IHs, resulting in less pain and faster recovery; however, its learning curve requires longer and more complex training than that for open surgical techniques. A greater number of supervised operations is necessary to achieve technical proficiency, which is one of the major obstacles to widespread adoption. Insufficient surgical training is associated with complications.^(1)^

Currently, various training methods are available, with the primary ones being cadaver dissection, virtual reality simulators, models synthesized from biological and/or synthetic materials, and 3D printed models. Utilizing cadavers in training would be most adequate for achieving anatomic similarity; however, this method is costly. The latest virtual simulators have the advantage of providing tactile feedback for surgery, but they are also expensive.

Two models were introduced in 2001: the Guildford MATTU TEP hernia model^(9)^ and the molded rubber hernia simulator.^(10)^ They are made of rubber and provide good anatomical representation, but they are expensive and difficult to transport. Other lower-cost models using photographs or recycled materials have emerged. However, they do not effectively represent the inguinal region as seen in a laparoscopic video view.^(11,12)^ In 2011, the McGill Laparoscopic Inguinal Hernia Simulator^(13)^ was created. It is a less expensive model that uses simple materials to simulate all the steps of an MIS. The model was evaluated using the Global Operative Assessment of Laparoscopic Skills-Groin Hernia. Surgeons trained on the model obtained lower rates of intra- and post-operative complications,^(14)^ demonstrating that these models can reflect an improvement in clinical practice.^(15)^

The dissemination and popularization of 3D printers have facilitated the development of more realistic and reproducible models without imposing significant financial burdens. Therefore, we opted to use a 3D printer to recreate the anatomy of the inguinal region with utmost similarity, ensuring cost-efficiency and ease of replication, as it only requires printing the file.

The 3D model used in this study was evaluated for realistic anatomical simulations and tissue manipulation. In contrast to biological tissue models, as presented by Ivakhov et al.,^(7)^ our model showed less sensitivity to dissection while maintaining a higher level of anatomical representation. Furthermore, our model is easier to manipulate, as performing a greater number of simulations using the same model is possible. In contrast, the biological model requires the preparation of pig stomachs for each simulation.

Considering training cost is necessary, as it is important for making dissemination feasible. Models are strategically less expensive; however, most importantly, they focus on the identification of specific structures, such as epigastric vessels, ductus deferens, and gonadal vessels, and the best anatomical representation of the inguinal region.^(7,11-13)^ The only previous model that used 3D printing recreated bone structures to simulate the pneumoperitoneum without reconstructing the inguinal region.^(8)^ We believe that this is the key difference in this new model.

Our objective was to develop an inexpensive model, priced at approximately 50 American Dollars (50 USD), that accurately replicated the anatomical features encountered during surgery, specifically recreating the inguinal region as viewed in video laparoscopy using 3D modeling. Twenty-eight surgeons underwent training with our model, seven of whom specialized in the treatment of abdominal wall hernias.

Based on simulations and questionnaire responses, the model was considered simple, realistic, and capable of precisely simulating inguinal anatomy for training purposes. Our 3D model, similar to the one developed by Nishihara et al.,^(8)^ offers a cost-effective and reusable training solution. Both projects demonstrated good acceptance of their implementation in the medical residency curriculum.

Furthermore, our study indicated good model acceptance despite limitations in the methodology employed. The questionnaire used to evaluate the model’s opinion did not assess the surgical technique. In addition, no comparisons were made with other existing models.

In laparoscopic IH surgery, we use little energy and more dissection movements, which can be simulated in the model. However, we did not foresee the use of electrosurgery, which can be considered a disadvantage of the model. Further studies are necessary to evaluate other important variables such as the cost-benefit relationship, reproducibility, required training time, and learning curve impact.

## CONCLUSION

Our inexpensive and reusable model was considered simple, realistic, and capable of precisely simulating inguinal anatomy for training purposes.
